# Considerations for legal, ethical, and effective practice in dementia research

**DOI:** 10.1093/braincomms/fcae211

**Published:** 2024-06-14

**Authors:** Michael C B David, Martina Del Giovane, Danielle Wilson, Trevor Truman, Jonathan D Huntley, Mehrunisha Suleman, Alexander Ruck Keene, Michael Parker, David J Sharp, Paresh A Malhotra

**Affiliations:** UK Dementia Research Institute Care Research and Technology Centre, Imperial College London, London W12 0BZ, UK; Brain Sciences, South Kensington, Imperial College London, London SW7 2AZ, UK; UK Dementia Research Institute Care Research and Technology Centre, Imperial College London, London W12 0BZ, UK; Brain Sciences, South Kensington, Imperial College London, London SW7 2AZ, UK; UK Dementia Research Institute Care Research and Technology Centre, Imperial College London, London W12 0BZ, UK; UK Dementia Research Institute Care Research and Technology Centre, Imperial College London, London W12 0BZ, UK; Division of Psychiatry, University College London, London W1T 7BN, UK; Faculty of Health and Life Sciences, University of Exeter, Exeter EX1 2LU, UK; Ethox, University of Oxford, Oxford OX3 7LF, UK; Global Studies Center, Gulf University for Science and Technology, 73F2+GV4, Masjid Al Aqsa Street, Mubarak Al-Abdullah, Kuwait; Barrister, London WC2A 1DD, UK; Visiting Professor, Dickson Poon School of Law, King’s College London, London WC2R 2LS, UK; Visiting Senior Lecturer, Institute of Psychiatry, Psychology & Neuroscience, King’s College London, London SE5 8AF, UK; Ethox, University of Oxford, Oxford OX3 7LF, UK; UK Dementia Research Institute Care Research and Technology Centre, Imperial College London, London W12 0BZ, UK; Brain Sciences, South Kensington, Imperial College London, London SW7 2AZ, UK; UK Dementia Research Institute Care Research and Technology Centre, Imperial College London, London W12 0BZ, UK; Brain Sciences, South Kensington, Imperial College London, London SW7 2AZ, UK

**Keywords:** ethics, law, academia, capacity, consent

## Abstract

Dementia represents a potentially overwhelming health burden, both for the UK and worldwide. Addressing this fast-growing issue is a key priority for the government, health service and the public. Advances in care including the development of efficacious disease-modifying, and eventually curative, treatments can only be achieved through effective dementia research. Specifically, research directly involving participants with dementia is essential to further understanding. However, working with cognitively impaired participants with and without capacity to consent to research presents unique ethical and legal challenges. For clinicians and scientists on the frontline of dementia research, scenarios frequently arise that pose such challenges. A lack of guidance for a consistent approach in navigating these scenarios limits researchers’ ability to proceed with confidence. This represents a threat to the rights and wishes of research participants as well as the field at large, as it may lead to studies being unnecessarily terminated or, worse, poor practice. In this article, we take a multiprofessional approach, informed by carer input, to these issues. We review the relevant ethical and legal literature relating to the conduct of non-interventional research studies in patients with dementia. This includes a thorough recap of the Mental Capacity Act (2005), which provides a legal framework in England and Wales for conducting research with participants who lack capacity to consent. We also discuss the important, but sometimes incomplete, role of research ethics committees in guiding researchers. We then present and discuss a series of case vignettes designed to highlight areas of incomplete coverage by existing governance. These vignettes describe theoretical scenarios informed by our own real-word experiences of encountering ethical issues when conducting dementia research. They include scenarios in which participants demonstrate varying degrees of understanding of the research they are involved in and ability to communicate their wishes and feelings. Building on these vignettes, we then provide a checklist for researchers to work through when presented with similar scenarios. This checklist covers the key ethical, legal and practical considerations that we have argued for. Taken together, this article can act as a guide, previously lacking in the literature, for colleagues in the field to enable much needed ethical, legal and effective research.

## Teaser

Dementia research involving participants with and without capacity presents unique ethical and legal challenges. David *et al*. review the relevant ethical and legal literature and then present and discuss some case vignettes that highlight such challenges. They then provide a novel checklist for researchers to work through when presented with similar scenarios.

## Graphical Abstract

**Graphical Abstract:**
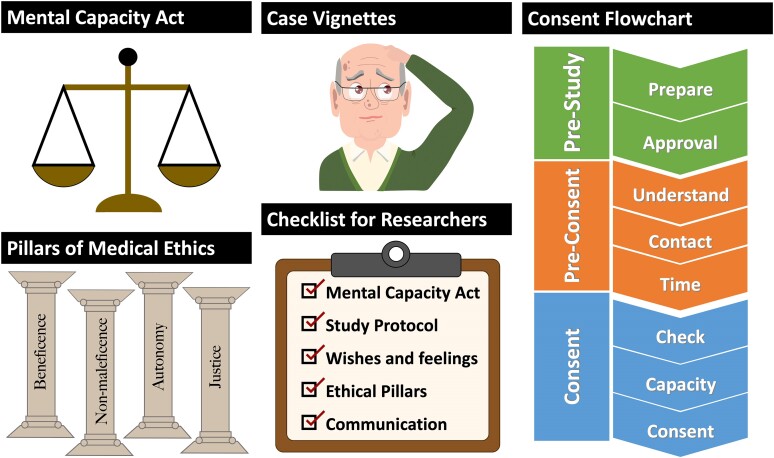

## Introduction

In the UK, and globally, an aging population is leading to dementia being, or fast becoming, the predominant public health issue.^1^ Despite the UK government’s intention for the country to become a ‘world leader’ in fighting dementia,^2^ there is less research in this area compared to other fields of medicine.^3^ While underfunding is one cause,^3^ it should be acknowledged that including adults who lack capacity in dementia research poses unique ethical challenges that require careful consideration.^4^

Our overall aim is to provide a practical, structured approach to decision-making when undertaking research with individuals who have significant cognitive impairment. The approach we set out is focused on ethical dilemmas that arise during ‘non-interventional’ studies in which participants may or may not have capacity, and their capacity may vary throughout the course of their participation. This work is provided for in England and Wales under specific conditions under the 2005 Mental Capacity Act (MCA).^5^ Participants’ attitude towards the study may also change. They may not always show enthusiasm and may sometimes express some concerns or reticence in participating.

This paper focuses on the ethics of dementia research that has no possibility of therapeutic benefit. Such work has huge utility to the field in both understanding the pathology but also enabling progress towards new treatments, ultimately tested in ‘clinical trials’. Large cohort studies, imaging databases and research collaboration platforms are all underpinned by research of this nature (see naccdata.org, adni.loni.usc.edu, dian.wustl.edu and dementiasplatform.uk for examples). As such, the research discussed in this article is distinct from clinical trials, which raise unique issues that are too numerous and complex to also give due consideration here. Such issues include, but are not limited to, the following: the need to ensure equipoise—a state in which the researcher genuinely does not know which arm of the trial is more beneficial^6^; the ethical intricacies of working with a clinical group, such as in dementia, where there are limited or non-existent therapeutic options and the state of desperation that may induce^7^; questions of ‘beneficence’ and ‘justice’ with regard to the potential unfairness in ‘not’ including eligible subjects in the trial^7^; and also the added issues related to the loss of capacity during a trial and how advance directives should and should not apply in the case of emergent benefit or harm from a clinical intervention.^7,8^

The authors of this article have diverse experience and expertise across the fields of dementia, ethics and law. We are made up of clinicians including academic neurologists, an academic psychiatrist and a junior doctor, as well as a neuroscientist, a barrister with expertise on capacity, an expert by experience as a carer and husband of an individual living with dementia and medical ethics experts including a research ethics committee (REC) chair and two bioethicists. The key terms used throughout are defined in a glossary (Box 1).

Box 1 **Glossary for key terms used. A list of key technical legal, research and philosophical terms used and their definitions**

**Table:** 

Capacity	The ability to make decisions as per the MCA. It is decision and time specific.^5^
Consent	The agreement to undergo treatment or participate in research (Fig. 2). Legally, it must be voluntary and informed. It can only be provided by a person with capacity.
Assent	An ethical concept of affirmative agreement (e.g. to participate in research), whereas ‘dissent’ is as an objection to participation. It requires a level of understanding less than that required for valid consent and the ability to indicate a meaningful choice.^9^
Authenticity	The congruence between a person’s wishes and values, and a decision made by them or on their behalf.^4^
Beneficence	A principle capturing the moral importance of benefiting others.^10^
Non-maleficence	A principle capturing the moral importance of avoiding causing harm to others.^10,11^
Autonomy	A principle capturing the moral importance of respecting the values and wishes of a person who has (or has had) adequate understanding and freedom to express them.^10,12^
Justice	The moral principle calling for benefits and harms to be distributed fairly. It can be subdivided into distributive justice, rights-based justice and legal justice.^11,13^
Moral agent	A person or entity who can make moral judgements and perform moral acts.
Virtue ethics	The branch of moral philosophy that concerns character traits a person should aspire to.^14^
Deontology	The view that rules, principles, codes of conduct etc. determine whether an action is right or wrong.^15^
Non-interventional study	A research study that, as opposed to a ‘clinical trial’, does not involve the formal evaluation of a therapy (pharmacological or non-pharmacological).

### The case for dementia research

An annual global burden of 1.6 million deaths and 28.3 million disability-adjusted life-years^16^ makes the need for dementia research an ethical imperative. The deficit between the resources supporting dementia research, and the burden of the problem, needs urgently addressing. The World Health Organization’s (WHO) 2022 dossier, ‘A blueprint for dementia research’, describes research and innovation as ‘integral parts of the global response to dementia’.^17^ The 2013 G8 summit set a target of identifying a cure or disease-modifying therapies by 2025, and 10 years on, we may be on the cusp of such breakthroughs, thanks to global research efforts.^18^ However, there is still some way to go,^19^ and dementia is currently the leading cause of death in England.^20^ Importantly, one of the WHO’s stated reasons that ‘most countries are far from reaching the adopted targets’ with respect to the public health response to dementia is a lack of skills in ethical research practice.^17^ In fact, healthcare workers in England and Wales do lack understanding of the relevant legislation.^21^ The WHO makes clear that transparency of regulatory frameworks and the ‘creation of ethically and morally sound guidelines’ would expedite ‘life-changing scientific advances’.^17^ With this paper, we hope to contribute to this particular need.

### Building a framework for ethical practice

First, we remind readers of the relevant sections of the MCA (Box 2) and go on to present a set of case vignettes highlighting theoretical scenarios informed by our own experiences, in which ethical issues arise during dementia research. These vignettes are designed to put these issues into a real-world context. We will discuss how one might navigate these issues with the aim of completing the study with a sound ethical approach complying with the law.

Box 2 **MAC recap. A reminder of the relevant sections of the mental capacity act relating to capacity assessment and conducting research with participants who do not have capacity to consent**

Here we recap the MCA,^5^ which constitutes the relevant legislation governing the assessment of capacity in England and Wales and is accompanied by a statutory Code of Practice^22^ providing guidance on how it should be used (see www.capacityguide.org.uk for guidance on assessing capacity). Section 51 of the Adults with Incapacity (Scotland) Act 2000^23^ and the Mental Capacity Act (Northern Ireland) 2016^24^ represent equivalent legislation in the other devolved nations. For simplicity, from here on, we concentrate on the England and Wales MCA.

Within the MCA, people without capacity to make a certain decision are those who, because of an impairment of or disturbance in the functioning of their mind or brain, are unable:

1. to understand the information relevant to the decision,
2. to retain that information,
3. to use or weigh that information as part of the process of making the decision, or
4. to communicate their decision.

The MCA requires that approval for research involving adults without capacity is contingent on the research:

- not being able to be carried out as effectively on exclusively those with capacity,
- having the potential to benefit the participant without disproportionate burden, and being intended to provide knowledge of the causes or treatment of, or of the care of persons affected by, the same or a similar condition,
- or, if without the potential to benefit, the risks are negligible and that anything done to, or in relation to the participant will not—
  - interfere with their freedom of action or privacy in a significant way or
  - be unduly invasive or restrictive.

When involving participants without capacity to consent to the research, the MCA: Code of Practice advises the researchers take reasonable steps to identify someone to consult. Such a consultee gives advice on what the participant’s wishes and feelings would be if they were able to consent. These wishes must be respected, but a consultee cannot provide consent on the participant’s behalf.^22^

Note that the research discussed here is of a nature described in c), and therefore, the burden of participation must not outweigh any potential (non-therapeutic) benefit or otherwise be negligible.

Second, we review the law in England and Wales and governance in the area of non-therapeutic dementia research. We highlight that despite providing broad guidance, they do not offer detailed ‘off-the-shelf’ answers for practice.

**Figure 1:**
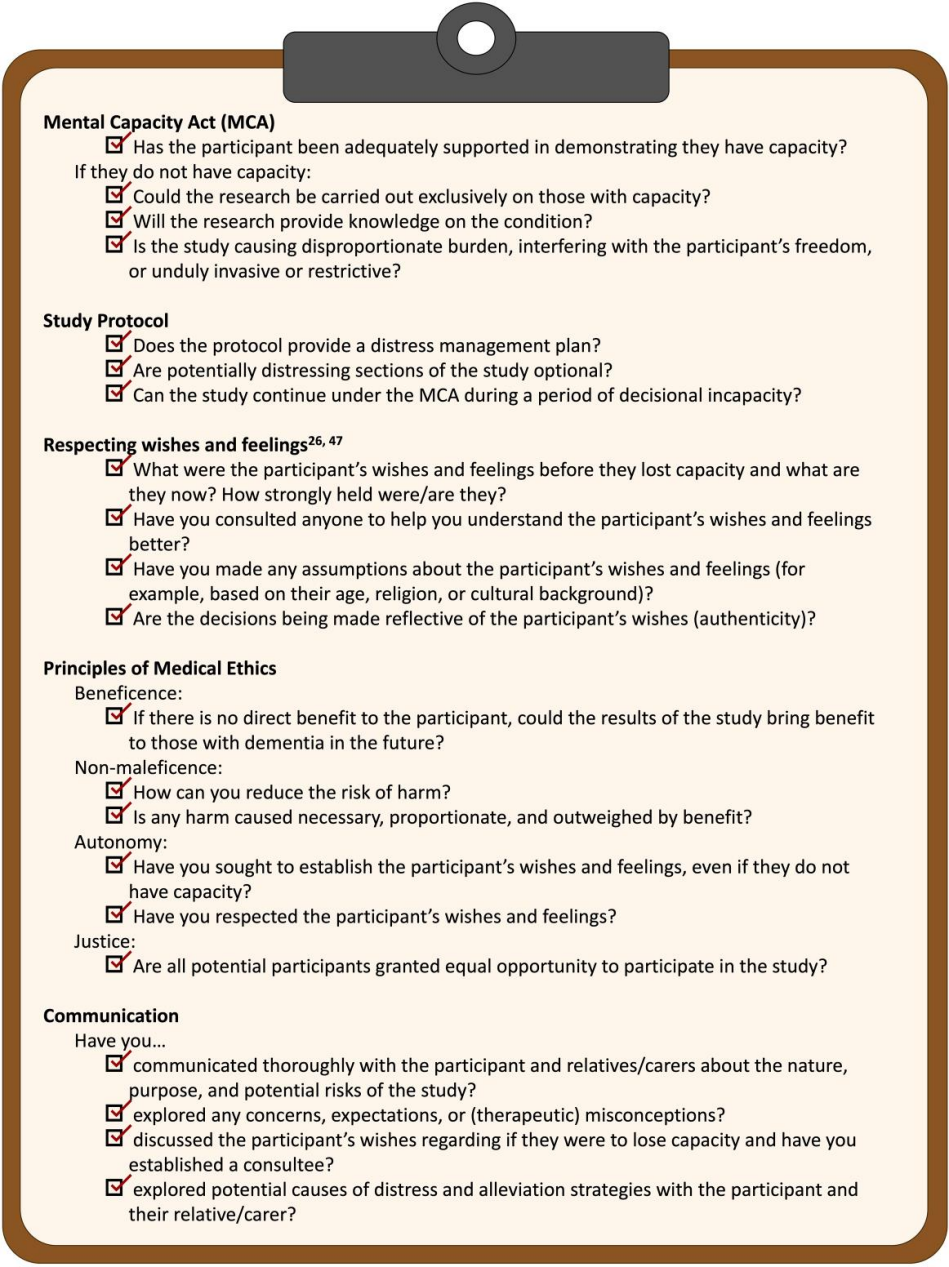
**Dementia research ethics checklist.** A list of the key considerations for managing ethical issues that may arise when conducting dementia research. By working through this checklist, researchers should be able to think through and answer the important questions related to any issues they may face. This will help them to proceed in a way that is ethical and lawful and provides the best chance of the participant completing the study. Readers outside England and Wales should consider that certain points may have to be adjusted in order to comply with the appropriate legislation and ethical requirements in their jurisdiction. MCA, Mental Capacity Act.

Third, we propose potential solutions for overcoming the shortcomings in the law as described above and distil these discussion points down to a checklist (Fig. 1) that researchers in the field can work through when presented with similar issues to those we describe. This will hopefully act as a guide—we believe currently lacking in the literature—for those conducting research involving adults with cognitive impairment, including those that lack capacity.

## Case vignettes

As dementia researchers, we can encounter situations where existing legal and regulatory frameworks lack clarity, potentially leading to sub-optimal ethical practices and incomplete research. To address this, we present a series of vignettes based on our experiences in dementia research. We hope these vignettes ring true to readers working in the field and provide real-life context for the subsequent discussion points. After each vignette, we encourage readers to consider how they would proceed, highlighting specific questions to be considered.

The nature of the studies in these vignettes, specifically the fact that they involve the study of disease neurobiology across the spectrum of severity, means they cannot be carried out effectively in people without dementia or exclusively in those with capacity. For all the vignettes, it is assumed that relevant study protocols, including the provision for involving individuals without capacity, have been REC approved. In addition, we assume that all staff have Good Clinical Practice training, have protocol training and are either clinicians or researchers with experience in working with people with dementia.

### Vignette A

A 78-year-old man with Alzheimer’s disease is taking part in a MRI study. The study’s aim is to understand how activities in the brain networks responsible for attention are altered in dementia and lead to everyday symptoms. He has a Mini-Mental State Examination (MMSE) score of 19/30. He was deemed to lack capacity to consent to take part in the study, and so his wife acted as his personal consultee prior to the scan. She stated that she believed he would like to take part. He made clear at this point that he was very happy to be contributing to dementia research, as it is something he strongly believes in. Before entering the MRI scanner, you explained to him that the scan would last about 1 h. He shows good understanding of what is going to happen and why. He is made comfortable in the MRI scanner and shows no sign of distress during the first 15 min. He then presses the emergency alarm, and you immediately stop the scan and attend to him. He appears anxious and says, ‘I don’t know what I’m supposed to be **doing**’. His wife tells you, ‘He just needs reminding of why he’s there. I know he wants to take part in the study’. How do you proceed?

Question:

1. Should you terminate the scan?
  Based on the emphasis in the participant’s wording, it is apparent that his concern is related to the fact he has forgotten why he is in the scanner—a situation that is inherently unfamiliar. This is unsurprising for a participant with moderate Alzheimer’s disease.^25^ In a scenario such as this, working with the consultee to inform and reassure patient is likely to be appropriate and necessary.
  According to the MCA [s.33(2)], an objection would prohibit you from continuing doing what he objected to; further, the participant must be withdrawn entirely from the research project if they indicate that is their wish [s.33(4)], with the threshold for withdrawal being set low. However, in the context of significant cognitive impairment, and an apparent genuine desire to participate in the study, we argue that, in this case, pressing of the buzzer does not equate to an objection to the scan or an indication that they wish to withdraw from the research. There certainly is a need to attend to the participant, explore the reason they pressed the buzzer and remind them of the purpose of the scan. If at this point, they make clear that they ‘object to what is being done to them’, or if they demonstrate enduring distress, it is clear that the legal and ethical thing to do would be to terminate the scan. By understanding that, in this scenario, the likely cause of distress is confusion, researchers should take the time to remind him of the purpose of him being there—that he is having a scan of his brain for the sake of research, something that he wanted to participate in. If this assuages his concerns, proactively reminding him of this at regular intervals will hopefully prevent further anxiety and distress. Clearly, it is important to ensure that the efforts to achieve clarification and understanding do not constitute persuasion. As such, the emphasis should be on explaining the purpose of the scan, in the hope that this alleviates his anxiety and means he does not wish to object, rather than the researcher convincing him to continue in the hope that he completes the study.
  Also, by law, the research should cease if his anxiety is causing greater than ‘negligible risks’, or if it ‘interferes with his freedom of action or is unduly restrictive’. While the MRI scan itself does not pose physical risks, there are ‘non-negligible risks’ associated with any anxiety caused. It is likely that reminding the participant of the purpose of the current activity will alleviate his anxiety. By providing him with a buzzer that he can use to stop the scan and be helped out of the scanner—and which he has demonstrated he is capable of using—you are avoiding the imposition of any undue restrictions. You may then consider it ethical, and defensible for MCA purposes, to continue. It is also important to ensure that there are no signs of significant distress of a nature or degree (e.g. trying to climb out of the scanner, becoming aggressive or tearful) that would make it unethical and unlawful to proceed, which there are not in this case as described. You should consider all these elements and work in collaboration with the consultee, in this case his wife.

### Vignette B

An 82-year-old woman with vascular dementia enrols in a longitudinal MRI study involving two visits, 1 year apart. The aim of the study is to assess how the rate of change in size of the hippocampi relates to worsening cognitive performance. For the first visit, she is accompanied by her husband. She has an MMSE^26^ score of 22/30, is assessed to have capacity, gives her consent to participate and completes the first scan without any problems. A year later, she returns for the follow-up scan, this time accompanied by her son as her husband has passed away. Her MMSE score is now 18/30. At each visit, you re-assess capacity and she now does not have capacity to consent, and therefore, the son will act as consultee. She tells you she is very happy to continue with the second scan. Before commencing the scan, the son tells you he thinks the scanner is too loud and claustrophobic for her and he is worried she might find it very distressing. He says, ‘She has become a lot more anxious over the last year. I would hate to be in that machine. I don’t want her to do it’. How do you proceed?

Questions:

1. Can you proceed on the assumption that the participant’s consent to take part is still valid, in spite of her son’s concerns?
  The 2009 Nuffield Council on Bioethics report on dementia highlighted a ‘lack of clarity about the procedures to be followed if a person gradually loses capacity to consent to their ongoing involvement in a research project’.^3^ How might such clarity be achieved in this case? With respect to ‘clinical trials’, UK regulations state that their consent to participate remains valid.^27^ In the context of a ‘non-interventional study’, however, governed by the MCA, consent cannot survive the loss of capacity to give it; therefore, in this vignette, you cannot proceed merely because the participant provided consent previously. The participant could only continue to take part in the MRI study if it was explicitly approved by a procedure complying with the research provisions of the MCA. When doing so, the value of ascertaining the wishes of the participant with dementia before they lose capacity, which has been highlighted by the Medical Research Council,^28^ should be kept in mind from the outset of the study. Wishes expressed prior to a participant losing capacity are extremely useful in understanding how best to proceed during a period of decisional incapacity. Taking this information forward requires researchers and consultees to balance multiple factors, including the nature and strength of previously expressed views at a time when the participant had capacity, as well as the consequences of respecting those views.^29^
2. To what extent does the consultee have ultimate authority in a scenario where the participant does not have capacity?
  The power of decision-making lies with the consultee, and the study should stop if the consultee is of the opinion that the participant’s ‘wishes and feelings would be likely to lead them to decline’ [MCA s.32(5)].^5^ The consultee has been initially unsure as to whether it would be reflective of her wishes for her to participate, but you have reason to believe it is her wish that she continues to participate. It seems sensible in this scenario to have a discussion with the son and the participant so that he has the opportunity to explore her wishes and for him to share his concerns. Note that here the son expressed that he himself would not like to be in the scanner, but this may not be true of the participant. To maximize the principle of ‘autonomy’, consultees and researchers should obtain what information is available about a participant’s preferences and objections (and hence gain their ‘assent’), when capacity to consent is lacking. While the participant no longer has capacity, they still have a critical role to play in this discussion.^30^ The participant may assuage the concerns of the consultee by stating that they are not concerned by the prospect of the scan—even if they do not meet the criteria for capacity to consent to the research project themselves. You may suggest, after discussions with the son and participant, and if the participant seems comfortable to continue, that the scan can be started, and that it could be stopped as soon as the participant objects.
  Ultimately, ‘if the consultee so advises, the participant must not take part and, if already taking part, must be withdrawn’^31^ [MCA s.32(5)]. However, in many cases following an inclusive conversation with the consultee and participant, it is likely that a way is found to enable the research to continue with the agreement of all parties.

### Vignette C

A 71-year-old man with frontotemporal dementia arrives at the laboratory for a PET scan. This scan will utilize a tracer that highlights activity in part of the brainstem known to be affected in frontotemporal dementia. He was driven by his son, and the journey took 2 h. The previous week, the participant with dementia had an MMSE score of 26/30, was assessed to have capacity and consented to the study and told you he was very much looking forward to it. On arrival at reception, the participant says, ‘I’m not sure I want to take part today. Can we reschedule for another day?’ The son is frustrated and says he thinks it would be a waste of everyone’s time to drive straight home again. He also tells his father that the researcher would incur a large, wasted cost if they had to cancel at the last minute and asks you confirm this. The tracer costs £2000 per dose, and you would not be able to recoup this cost. How do you proceed?

**Figure 2:**
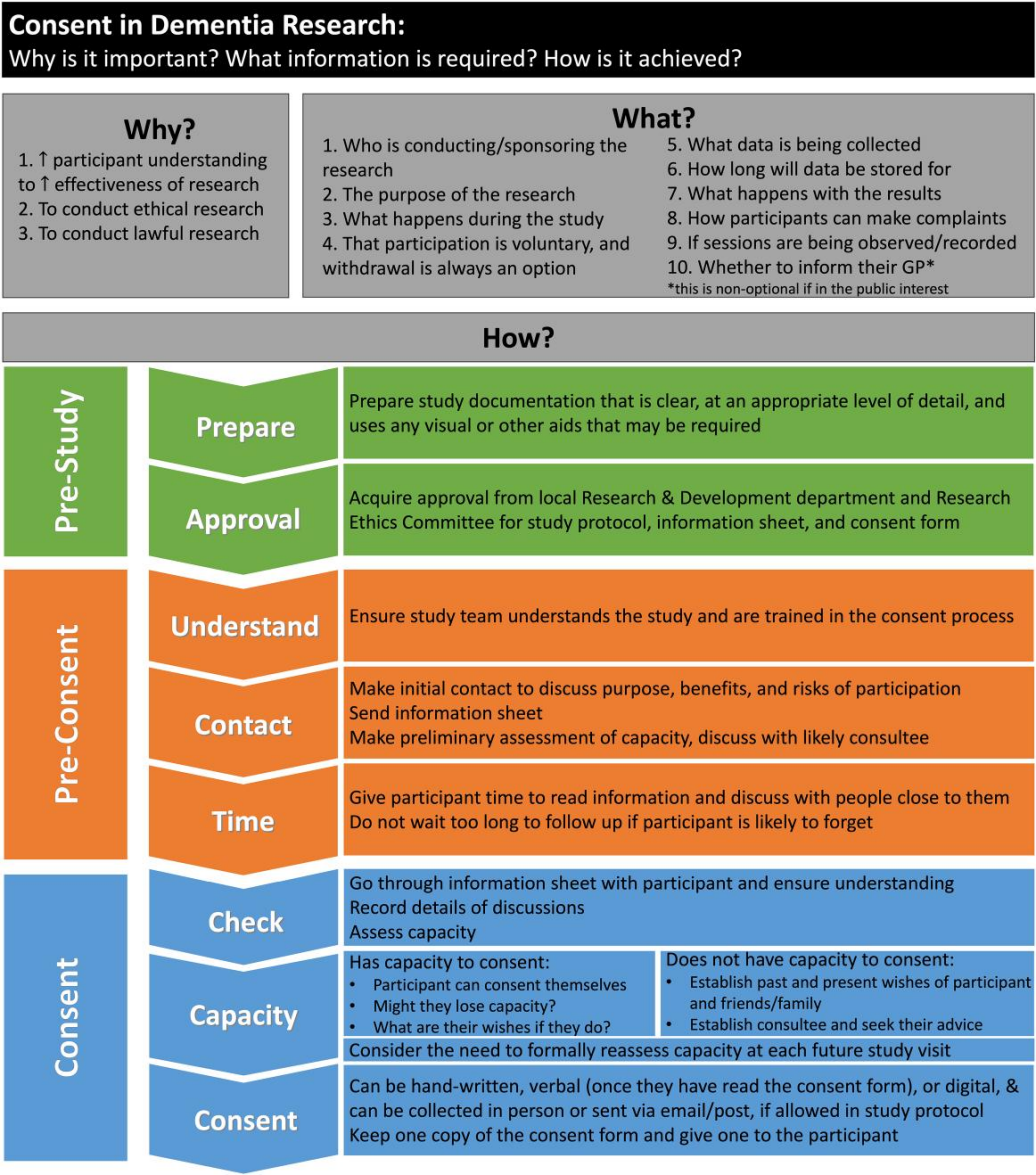
**Flowchart for the consent process in dementia research.** A summary of why conducting the consent procedure properly is important in dementia research and what information should be provided to the participants. Followed by the steps that should be taken in order to achieve effective, ethical and lawful acquisition of consent for participation in dementia research. Based upon guidance available online from the Health Research Authority,^34^ the General Medical Council^35^ and the UK government^36^ (see also Dewing^37^ for detailed guidance). GP, general practitioner.

Questions:

1. Does the participant have the information required to decide whether they would rather spend 4 h in the car without participating or go ahead with the scan?
  If the participant and his son were to return straight home, without the participant’s concerns being fully explored, this could result in an unsatisfactory scenario for all parties. In dementia research specifically, it is appropriate, and often necessary, to take additional time reiterating the nature and purpose of the study. In this vignette, helping the participant to recall and understand why they are there and what the day will involve may reignite their enthusiasm. Whereas in a study involving people without cognitive impairments this may seem unnecessary, here you may find that the participant’s misunderstanding or misremembering what they are there to do is a factor. The European Court of Human Rights has emphasized the need for particular vigilance before concluding that those with cognitive impairments have consented to clinical research.^32^ This suggests that the threshold for refusing to consent is lower than the threshold for consenting. However, to maximize the chance of the research being conducted, it is important to explore the participant’s views and concerns—specifically why they appear reluctant to proceed on this day—before cancelling their participation. This exploration must not constitute persuasion. Whatever the final decision, this approach will maximize autonomy and reinforce the informed consent of the participant.
  Notably, while it is pertinent to consider the interests of the son, who is likely to be frustrated by having to return home straight away, you must ensure that decision lies solely with the participant in this scenario, as they have capacity. Therefore, if the participant, after being reinformed about the research, still expresses a wish not to take part, it is clear that you must not proceed.
2. Should you disclose to the participant the fact that you will incur an irredeemable cost of £2000 if they choose to reschedule?
  It is essential that the participant does not feel coerced, as this is a threat to the consent being valid, and is equally important to it being capacitous (see Hawkins and Emanuel^33^ for a detailed discussion). There is a risk that by revealing the potential wasted cost, the participant will feel under pressure to participate on this day whereas they would otherwise choose not to. It is the researcher’s responsibility to account for costs such as these when budgeting for their project—pressuring individuals to not waste money is likely to lead to unethical decision-making.
  The principle of ‘justice’ is pertinent here. There are limited resources in dementia research, and you have an ethical obligation to use those resources fairly and to maximize benefit to the field. That being said, the costs incurred for each participant should not be a factor in how the researcher communicates with the participant. All participants with capacity must be allowed to make a free and informed choice and have the right to change their mind.
  This vignette also highlights the importance of considering how a study protocol can be flexible enough to facilitate the different requirements of each participant while maintaining scientific consistency. With the ultimate objective of facilitating participation, it is worth exploring having multiple study sites or providing transportation, for example, so as to limit the inconvenience for participants and their consultees. It would be unfortunate, and unethical, for this to be hindered by avoidable inconvenience.
  You have been asked by the son to confirm that a large cost would be incurred and there is a duty to answer truthfully—truthfulness is a cornerstone of ‘virtue ethics’ and ‘deontology’.^14^ It is important to keep the participant at the centre of the discussions here, and if asked directly, you should be honest that the costs cannot be recouped, but offer assurance that costs are entirely the responsibility of the research team. Both ‘virtue ethics’ and ‘deontology’ would also call for a sensitive approach in which one’s obligations to the participant were met. Honest, collaborative research is preferrable to lying, even if it the lie is to prevent them feeling coerced to participate. Hopefully, by taking time to provide the participant with the necessary information and allowing them time to come to the decision to participate, free from any pressure to do so, you will remove any doubt about the validity of their consent (Fig. 2).

### Vignette D

You are a junior doctor running an EEG study in Alzheimer’s disease. The project involves analysis of brain activity during rest and cognitive tasks, as a way of understanding more about how electrical activity in the brain is altered by the disease. There is no therapeutic benefit to participants. A 77-year-old participant with dementia with an MMSE of 25/30 is undergoing the EEG procedure having been assessed to have capacity and giving consent prior to starting. In a break between the (passive) resting-state and (active) cognitive task sections of the protocol, the participant says to you, ‘I’m not really sure how this cap on my head is going to make my memory better doctor, but I sure hope it does!’. How do you proceed?

Questions:

1. Does the statement suggest the participant does not have capacity as first thought?
  This appears to be an example of ‘therapeutic misconception’, which occurs when ‘a research subject fails to appreciate the distinction between the imperatives of clinical research and of ordinary treatment, and therefore inaccurately attributes therapeutic intent to research procedures’.^38^ Participants with dementia are particularly vulnerable to this phenomenon^39^ and, when it occurs, it poses questions about their capacity to participate. It has been argued that when the context (the clinical environment, the presence of a doctor, etc.) is strongly reminiscent of the participant’s experience of clinical care, it is unsurprising if this phenomenon occurs. Relatedly, in this context, there is an intrinsic power imbalance between doctor/scientist and participant, which must be considered here as a factor contributing to the risk of coercion.
  Regarding this question, we must go back to the MCA s.3(1) for the definition of capacity.^5^ To be assessed to have capacity, she must understand, retain and use or weigh the information and then communicate her decision. We need to then apply these four components in turn to the participant’s handling of the information on the nature and purpose of the study, and the distinction between clinical care and research.
  Before determining whether the participant has understood this information, the researcher may want to reiterate the fact that the EEG is not a treatment. Once it has been established that this information was made clear in the first instance, and that the participant has understood it, you must test whether she retains this understanding over a reasonable period of time. Next, while they may have understood and retained the information that the research project is ‘not’ part of their clinical care, if they still wrongly assume potential therapeutic benefit, it could be argued that they have not effectively used or weighed the information. Hence, they would not have capacity as per the third requirement of MCA s.3(1). In fact, as others have put it, participants’ ‘willingness and motivation to participate in research is not always based on a rational weighing of risks and benefits’, in which case they would not meet the criteria for capacity. Instead, their willingness and motivation may be driven by a faith that doctors know what is best and that any co-operation is likely to bear clinical benefit.^40,41^ In the case of non-interventional studies such as this one, with no such clinical benefit, this constitutes a misconception.
2. Is there an ethical obligation to correct her misconception that the study may be of therapeutic benefit, and if so, how is this best achieved?
  Therapeutic misconception is a threat to good ethical research, as participants should not be taking part under the misconception that they may be receiving treatment. Also, therapeutic misconception has the potential to confound the research, particularly if the intention is to study brain activity at ‘rest’. A participant undergoing a procedure that they believe may be treating their dementia is unlikely to be in a cognitive ‘resting state’. Therefore, the answer is yes, there is an obligation to correct the misconception.
  It has been argued that the researchers themselves can be prone to confusion as to where boundaries exist between research and clinical care, which may compound the issue.^42^ For example, in MRI studies, researchers may explain to participants that the images will be reviewed by a radiologist, and they will be informed of the results. This is primarily for management of incidental findings, but a participant would be forgiven for seeing this as an opportunity to access a diagnostic test as part of a clinical pathway. Therefore, it is important in scenarios such as described in this vignette that the researcher is clear in their own mind about the distinction before reiterating it to the participant. This would enable the communication of a clearer, more consistent message to the participant with respect to the fact that this procedure is not therapeutic. The participant can only be assessed to have capacity to consent if they understand this distinction specifically.
  We also suggest that communicating the results of the research to the participants, at the individual and study level, can reiterate the scientific, rather than therapeutic, nature of their contribution and can remind them of the value of their participation.

### Vignette E

An 88-year-old man with dementia with Lewy bodies is attending the lab for a blood test as part of a research study. The research of which this blood test is a part will hopefully allow its translation into clinical practice. He has an MMSE of 16/30 and does not have capacity to consent to the research, so his wife has acted as a personal consultee and advised he would wish to participate. The participant has been agitated since arriving at the lab and his wife tells you, ‘He slept poorly and has not been his usual self today’. The participant is on the clinic bed and when he sees you prepare the needle for venepuncture, he becomes concerned and says, ‘I don’t think I need a blood test because I already had one at another hospital this morning!’ His wife tells you he has never had a problem with needles before and that he has not had a blood test recently. How do you proceed?

Questions:

1. What is the significance of the wife’s information that the participant did not have a blood test that morning?
  The wife’s information suggests that the participant’s claim that he had a blood test this morning may not be the root cause of his reluctance. This is a common scenario in dementia research. Participants with significant cognitive impairment may be unable to understand the reason they feel anxious and are likely to attribute this to a false memory. False memories and, relatedly, confabulations are known to occur in dementia.^43-45^ False memories are more likely to be seeded at times of emotional stress,^43^ and it seems likely that stressful situations like the one in this vignette could also induce the calling upon of false memories.
  Ethically, a procedure should be paused if the participant ‘dissents’, irrespective of the reason to do so. However, there is also an ethical obligation to attempt to alleviate the participant’s distress, in the hope that, but not dependent on, the possibility that this will enable the research to continue. Given the information provided by the wife, it is apparent that the participant’s statement is a confabulation (or false memory), and this has implications for how it is addressed. Specifically, the researcher and consultee should explain to the participant that they did not have a blood test this morning, and that the purpose of this test is for research and, therefore, is different to any previous tests he may have had in the hospital. But also, the researcher and consultee should recognize that the expression of a confabulation may be the result of an underlying anxiety in this scenario, the true cause of which should be explored and alleviated.
2. Is there an obligation to withdraw the participant from the study based on their reaction to seeing the needle?
  The participant has made a clear statement that they do not want the blood test. However, it is possible that this does not reflect his lasting view, but rather a momentary expression of anxiety. He does not have capacity to participate in the research and therefore you must consider whether the blood test would be deemed ‘unduly invasive’ or pose a non-negligible risk in light of his apparent distress. In view of these considerations, it seems reasonable to explore further the reasons for his distress and assess whether it is sustained, as well as the views of the consultee, before making a decision to withdraw him entirely. Once this is done, the most appropriate course of action may be to rearrange the blood test for another day, if the consultee agrees.

## Limitations of the MCA

The MCA is fundamental both to lawful action and to good practice in England and Wales and should always be given diligent consideration by researchers. We argue, however, that within clinical dementia research, the MCA and Code of Practice are not always sufficient to dictate ethical practice and that there are circumstances, such as those described in the vignettes above, in which more specific guidance would be helpful. These circumstances may involve people with capacity, those who lack capacity or have fluctuating capacity and those in whom the presence or lack of capacity is unclear.

### Participants with capacity to consent to the research

In circumstances in which a dementia research participant has capacity, best and lawful practice should in theory be relatively uncomplicated. In this situation, the participant can be supported to make their own decision about what research they want to participate in including if/when they wish to withdraw. In the case vignettes presented above, we highlighted how ethical problems can, however, still arise in situations where research involves participants with dementia, even if they have capacity. For example, where the participant appears misinformed about the study, they are consenting to (see ‘Vignette C’ and ‘Vignette D’).

### Participants without capacity to consent to the research

Participants with even mild–moderate dementia often lack capacity to consent to the research.^46^ It would be unethical, and a threat to their human rights,^47^ to prevent this group from participating in research. Participants without capacity should be able to access the benefits that come with participation in non-interventional studies, even if they are not ‘therapeutic’—for example the enjoyment and/or sense of fulfilment that often comes with participation.^3^ The MCA does not prohibit the inclusion of those without capacity to consent to research, but over-cautious interpretation of the law can potentially prevent such individuals who express a desire to participate, from doing so, reducing their autonomy and de-prioritizing their wishes^40^ (see ‘Vignette B’).

In addition to the importance of involving these participants for their own sake, it is vital from a societal perspective. We must ensure that people who lack the capacity to consent to research studies, including those with advanced dementia, are involved as this will further scientific understanding of the disease processes up to and including these latter stages and inform clinical trial design,^29^ in turn conveying benefits for others with advanced dementia.^4^ Therefore, there is a need for ethical and legal avenues, accompanied by clear guidance, for conducting research in dementia patients without capacity.

### Uncertain capacity

In some cases, it may not be possible to make a reliable decision regarding a research participant’s capacity to make a specific decision.^48,49^ In fact, it is not always easy for clinicians to define patients clearly as having capacity or not.^40,46^ Capacity varies within as well as between individuals, depending on environmental, social and decision-specific factors.^50^ Also, cognitive performance measured using standard tools is insufficient to determine capacity, which complicates the assessment process.^46,50^ ‘Vignette D’ highlighted how the capacity of a participant who seems relatively cognitively intact may not hold up to scrutiny in certain contexts. Additionally, capacity in some people with dementia is prone to fluctuations, declines over time (as in ‘Vignette B’) and varies across individual decisions.^3,51,52^ Therefore, dementia researchers need to be able to approach the recruitment of participants lying anywhere along the ‘capacity spectrum’ in an ethical way.^46^

### Potential solutions to issues of capacity

#### Supporting the participant in the capacity assessment

To establish that a participant with dementia has capacity to consent to research is to empower them and maximize their ability to have their wishes realized. MCA s.1(3) implores assessors of capacity to take ‘all practicable steps’ to support the person being assessed.^5^ In their report on dementia, the Nuffield Council on Bioethics’ position is that ‘a person with dementia should receive all possible support to help them make their own decision about involvement in a particular piece of research’. It specifically advises researchers and RECs to ‘adapt the informing process in a way to enable, rather than to exclude, people with dementia’ (Fig. 2).^3^ Providing support in the way of adequate information, at an appropriate level of detail, with ample time for consideration is clearly crucial in dementia research. However, as HHJ Rogers^53^ ruled in a 2020 case regarding the capacity of a woman with intellectual disability, ‘there comes a point where support and encouragement becomes so integral to the decision making process that, in reality, the individual concerned is … simply carrying out the instruction of others rather than … making capacitous personal decisions’. While no specific criteria to determine that this is the case exist, dementia researchers should keep in mind that there may come a point at which the support required for a participant to use and weigh the information presented to them is to a degree that they are no longer demonstrating capacity as an independent agent. At that point, unless the research can be carried out under the specific research provisions of the MCA, it cannot proceed.

#### Respecting wishes and feelings in the absence of capacity

In relation to those who do not have capacity to consent, it can still be ethically meaningful to distinguish between the person who ‘assents’, and the person who ‘dissents’ (see ‘Vignette B’). This requires researchers to engage in a meaningful way with the participant and the evidence they have for their preferences.^4,40^ Establishing these preferences is key to respecting their autonomy and hence acting ethically. As Hope *et al.*^29^ argues, ‘A person with dementia may fail tests of capacity for almost any decision and yet may have wishes and desires and be able to express these, if not verbally then behaviourally. It would be in keeping with the value of respecting capacitous choices to give at least some weight to such desires’. This chimes with the MCA: Code of Practice that states researchers should ‘do whatever is possible to permit and encourage the person to take part, or to improve their ability to take part, in making the decision’.^22^

Another way to maximize the incorporation of participants’ wishes is by pursuing ‘authenticity’. This can be achieved, for example, through the assignment of a surrogate, or consultee, to advise the researchers (see ‘Vignette A’). The consultee’s task by s.32(4)(b) MCA is to seek to advise what the person’s wishes and feelings about taking part in the project would likely be if they had capacity (in relation to the question posed).^5^ Even if the consultee cannot give consent on the person’s behalf, their involvement provides a way in which—working together—the researcher and the consultee can achieve a maximally participant-centred approach (Fig. 2).

#### Navigating uncertain capacity

It is important to note here that, while such a spectrum exists in reality, adherence to the MCA requires a decision to be made as to whether a participant has capacity or not.^5^ The lack of a single tool to measure capacity reliably means those making the assessment must aim to have a legitimate process for carrying out and recording the outcome of an assessment.^50^ This allows them to come to a conclusion as to participants’ capacity that is consistent, justifiable and legally sound. What remains crucial, regardless of the conclusion reached, is that the researcher does not lose sight of the need to respect the core principles of medical ethics, including patient’s autonomy—by establishing and respecting their wishes as fully as possible.

## RECs

In order to comply with the MCA, medical research must be conducted in accordance with protocols that have been approved by an independent REC. RECs are responsible for safeguarding the rights, safety, dignity and wellbeing of research participants by giving opinions on whether proposed research is ethical and compliant with the law.^54^ They act to protect the relationship between researchers and participants by acting impartially in the interest of both parties. By providing advice to researchers, they can be a vital resource for achieving good, ethical research but also will defend the rights of participants, vetoing unethical proposals, ensuring clear communication to participants of their rights and that their needs are met.^55,56^ However, evidence regarding the effect of REC input into studies is not routinely evaluated and is therefore limited and inconclusive.^56-59^ Overall, we acknowledge the important role of RECs but that there are likely limits to their ability to ensure ethical practice. Therefore, we feel there is a need for additional guidance for researchers on how they themselves can ensure that they are conducting research with a consistent and sound ethical approach.

### Ethical practice beyond study protocols

As described in Faber Post and Blustein’s^60^  *2021 Handbook for Healthcare Ethics Committees*, RECs should ‘strive to develop ethics expertise … through education, policy development and consultation’. But the authors also state that ethics should be taken seriously by everyone rather than ‘off-loaded’ to a REC. We agree that good or poor ethical research cannot be the responsibility exclusively of an ethics committee. Researchers are themselves ‘moral agents’ and hence have the ability, and obligation, to make moral judgements and perform moral acts. As such, they must continue to develop skills to appraise and act in accordance with the ethical principles when conducting research—rather than relying on, and being limited by, what is stated in their protocol. As Johnsson *et al.*^61^ summarizes, ‘the efficacy of ethics review in safeguarding morally acceptable research depends on the moral competence and integrity of individual researchers’. In the vignettes presented above, we highlighted scenarios in which there is unlikely to be specific direction within a study protocol for how to act, and therefore, a degree of judgement is required by the researcher.

We also acknowledge that many frontline researchers may lack confidence in their own training for achieving best ethical practice. For effective implementation of researcher autonomy as described above, we advocate for greater inculcation of best practice by experienced colleagues and official bodies.^56^ Currently, only Good Clinical Practice certification, the agreed international standard for conducting clinical research, is mandated.^62^ Fortunately however, in the UK, the National Institute for Health and Care Research,^63^ local clinical research networks^64^ and the Health Research Authority^65^ all offer training that could be better utilized by the field. Alongside specific training, there should be an obligation for senior colleagues within research groups and clinical facilities to imbue a supportive and educational environment that instils in the local culture, an importance on well-informed, ethical practice. Good leadership includes reinforcing the importance of following regulations, overseeing day-to-day behaviours and creating a supportive environment where junior researchers can observe best practice from colleagues with greater experience.^66^

## A checklist to guide decision-making

Informed by the authors’ real-life experience of issues that arise in non-interventional dementia research studies, such as those described in the vignettes, we propose the following checklist (Fig. 1). Our intention is for researchers to work through this checklist when presented with an ethical issue in order to make a justifiable decision that is ethically and legally sound and that aids participation without coercion. This is founded in established ethical principles and builds on the solutions proposed above for navigating limitations in existing frameworks. This is not intended to provide definitive direction in the case of specific issues but to be generically applicable across a range of scenarios and should be applied upon a basis of good training and leadership.

## Conclusion

Conducting effective and ethical dementia research is challenging as illustrated by the vignettes above. The existing legal, ethical and governance frameworks are overlapping, multifaceted but also often insufficient. Situations arise that are not specifically prescribed for within legislation, considering a range of ethical principles can lead to conflicting conclusions, and RECs do not and should not have the ultimate authority over how a study is conducted.

In the context of studies such as those discussed here, in which there is no therapeutic component, ethical issues are commonplace (the ethics of ‘clinical trials’ in this population also warrants thorough, up-to-date exploration, with sufficient patient and public involvement). Researchers can feel pressure to collect data to achieve their own professional goals, to satisfy the stated requirements of a study timeframe, to comply with the demands of research primary investigators or supervisors and not to waste funding. They are also committed to benefitting people with dementia. However, they also know that there may be limited direct benefit to their participants. Therefore, there is a conflict between the desire to complete the research but an awareness that any harms caused are rarely justified.

We presented a series of case vignettes, based on the real-life experience of the authors working across the fields of dementia research and medical ethics. Subsequently, we discussed how researchers might approach the issues that arise in these vignettes, complying with the law and with guidance from established ethical and philosophical principles. We then discussed the MCA and role of RECs and highlighted their limitations, before suggesting some ways of overcoming these limitations. Finally, we have produced a checklist (Fig. 1) for researchers to work through when presented with ethical issues. By utilizing this checklist, colleagues can approach ethical dilemmas in a systematic manner and facilitate participation in much needed dementia research.

## Data Availability

Data sharing is not applicable to this article as no new data were created or analysed in this study.
